# Normal Values of Corrected Heart-Rate Variability in 10-Second Electrocardiograms for All Ages

**DOI:** 10.3389/fphys.2018.00424

**Published:** 2018-04-27

**Authors:** Marten E. van den Berg, Peter R. Rijnbeek, Maartje N. Niemeijer, Albert Hofman, Gerard van Herpen, Michiel L. Bots, Hans Hillege, Cees A. Swenne, Mark Eijgelsheim, Bruno H. Stricker, Jan A. Kors

**Affiliations:** ^1^Department of Medical Informatics, Erasmus University Medical Center, Rotterdam, Netherlands; ^2^Department of Epidemiology, Erasmus University Medical Center, Rotterdam, Netherlands; ^3^Julius Center for Health Sciences and Primary Care, University Medical Center Utrecht, Utrecht, Netherlands; ^4^Department of Cardiology, University Medical Center Groningen, Groningen, Netherlands; ^5^Department of Cardiology, Leiden University Medical Center, Leiden, Netherlands; ^6^Department of Internal Medicine, University Medical Center Groningen, Groningen, Netherlands; ^7^Department of Internal Medicine, Erasmus University Medical Center, Rotterdam, Netherlands; ^8^Health and Youth Care Inspectorate, Utrecht, Netherlands

**Keywords:** electrocardiography, heart-rate variability, normal values, heart-rate correction, children, adults, elderly

## Abstract

**Purpose:** Heart-rate variability (HRV) measured on standard 10-s electrocardiograms (ECGs) has been associated with increased risk of cardiac and all-cause mortality, but age- and sex-dependent normal values have not been established. Since heart rate strongly affects HRV, its effect should be taken into account. We determined a comprehensive set of normal values of heart-rate corrected HRV derived from 10-s ECGs for both children and adults, covering both sexes.

**Methods:** Five population studies in the Netherlands (Pediatric Normal ECG Study, Leiden University Einthoven Science Project, Prevention of Renal and Vascular End-stage Disease Study, Utrecht Health Project, Rotterdam Study) provided 10-s, 12-lead ECGs. ECGs were stored digitally and analyzed by well-validated analysis software. We included cardiologically healthy participants, 42% being men. Their ages ranged from 11 days to 91 years. After quality control, 13,943 ECGs were available. Heart-rate correction formulas were derived using an exponential model. Two time-domain HRV markers were analyzed: the corrected standard deviation of the normal-to-normal RR intervals (SDNNc) and corrected root mean square of successive RR-interval differences (RMSSDc).

**Results:** There was a considerable age effect. For both SDNNc and RMSSDc, the median and the lower limit of normal decreased steadily from birth until old age. The upper limit of normal decreased until the age of 60, but increased markedly after that age. Differences of the median were minimal between men and women.

**Conclusion:** We report the first comprehensive set of normal values for heart-rate corrected 10-s HRV, which can be of value in clinical practice and in further research.

## Introduction

Heart-rate variability (HRV) as measured on the electrocardiogram (ECG) is the variability of intervals between QRS complexes generated by sinus node depolarization in one continuous recording (Task Force of the European Society of Cardiology and the North American Society of Pacing and Electrophysiology, 1996). Many studies have indicated that reduced HRV is a strong, independent, and consistent risk factor for all-cause and cardiac mortality (Billman, 2011). HRV guidelines recommend that measurements be based on 5-min or 24-h ECG recordings, but 10-s ECGs are more commonly made during routine medical care and are faster, cheaper, and more patient-friendly than longer ECG recordings. Furthermore, even though it is not possible to determine frequency-domain measurements nor some of the time-domain measurements on a 10-s signal, it is possible to obtain the two most commonly used time-domain measurements: the standard deviation of the normal-to-normal RR intervals (SDNN) and the root mean square of successive RR-interval differences (RMSSD) (Task Force of the European Society of Cardiology and the North American Society of Pacing and Electrophysiology, 1996). It has recently been demonstrated that there is substantial agreement between 10-s and 4–5-min pulse-wave recordings for RMSSD, and to a lesser extent for SDNN (Munoz et al., 2015). As a matter of fact, time-domain HRV markers as measured on 10-s ECGs have been associated with heart failure (Rautaharju et al., 2006), cardiac mortality (de Bruyne et al., 1999), and all-cause mortality (Dekker et al., 1997) in population-based studies. In one of these studies (de Bruyne et al., 1999), both high HRV and low HRV were associated with adverse outcomes. Thus, 10-s HRV seems a possible tool in epidemiological research and risk assessment.

Heart-rate variability is known to have a strong, inverse relationship with heart rate (Coumel et al., 1994; Tsuji et al., 1996; Monfredi et al., 2014; Sacha, 2014; Gasior et al., 2015). It has been suggested by Monfredi et al. (2014) that this relationship is exponential, and that HRV parameters should be exponentially corrected for heart rate. However, the aforementioned studies either did not adjust for heart rate, or did so applying a linear adjustment. As heart-rate itself is a strong risk factor for cardiac morbidity and mortality (Greenland et al., 1999; Fox et al., 2013), the results of the earlier studies might have been confounded.

Knowledge of normal values of heart-rate corrected HRV markers from 10-s ECGs would allow to derive well-grounded thresholds for continuous variables in risk models and may be useful in establishing diagnostic criteria. A number of studies have reported normal HRV values for 5-min (Nunan et al., 2010; Kim and Woo, 2011; Seppala et al., 2014) and 24-h (Umetani et al., 1998) ECG recordings, but only one recent study reported normal values for 10-s ECGs (O’Neal et al., 2016), without investigating the age-dependence of HRV. Moreover, none of these studies applied heart-rate correction. Therefore, in this study we determine heart-rate corrected normal values for HRV as derived from 10-s ECGs across all age groups.

## Materials and Methods

### Study Populations

In this study we combined data from five population studies conducted in the Netherlands. The 10-s 12-lead ECGs from these studies were digitally recorded and stored at sampling rates of at least 500 Hz, up to 1200 Hz in the pediatric group. ECGs were recorded with the subjects in supine position. All data were anonymized.

(1) Pediatric Normal ECG Study (Rijnbeek et al., 2001). The population of this study consists of 1,912 children, their ages ranging from 11 days to 16 years. The children were recruited in the year 2000 at three child health centers, three primary schools, and one secondary school in the city of Rotterdam. The children’s height and weight, measured before ECG recording, corresponded well with the Dutch growth standard. ECGs were recorded with a portable PC-based acquisition system (Cardio Control, Delft, Netherlands).

(2) The Leiden University Einthoven Science Project (Scherptong et al., 2008). The population of this study contains 787 medical students of Leiden University. The ages of the participants range between 17 and 29 years, and all attested to be in good health. The ECGs were recorded from 2005 until 2007 with Megacart electrocardiographs (Siemens, Erlangen, Germany).

(3) The Prevention of Renal and Vascular End-stage Disease (PREVEND) Study (de Jong et al., 2003). This study, which started 1997, has as its goal to investigate the natural course of microalbuminuria and its relation to renal and cardiovascular disease in the general population. The PREVEND population consists of 8,592 participants aged 28–75 years, from the city of Groningen. Medical records, including medication use, were available for all participants. ECGs were recorded with CardioPerfect equipment (Welch Allyn Cardio Control, United States).

(4) The Utrecht Health Project (Grobbee et al., 2005). This ongoing study started in 2000 in Leidsche Rijn, a newly developed residential area of Utrecht. All new inhabitants were invited by their general practitioner to participate. The population of this study consists of 6,542 participants. Written informed consent was obtained and an individual health profile was made by dedicated research nurses. Baseline assessment included physical examination, ECG, blood tests, and interview-assisted questionnaires. Pharmacy records were used to obtain medication use. ECGs were recorded with CardioPerfect equipment (Welch Allyn Cardio Control, United States).

(5) The Rotterdam Study (Hofman et al., 1991). This study, which started in 1990, investigates determinants of a number of age-related disorders in an elderly population, prominently among them cardiovascular disease. The Rotterdam Study population consists of 10,994 inhabitants of Ommoord, a suburb of Rotterdam, aged 55 years or older. Participants were visited at home for an interview and were subsequently examined at the research center. Detailed information was collected on health status, medical history, and medication use. ECGs were recorded with an ACTA electrocardiograph (Esaote, Florence, Italy).

From these five populations, totaling 28,827 participants, we selected a subgroup of participants with no indication of cardiac disease. Reasons for exclusion were a history of myocardial infarction, heart failure, coronary bypass surgery, coronary angioplasty, or pacemaker implantation. Other exclusion criteria were hypertension and diabetes mellitus. Hypertension was defined as a systolic blood pressure ≥160 mmHg or a diastolic blood pressure ≥100 mmHg or use of antihypertensive medication, including use of beta-blockers. Diabetes mellitus was defined as a non-fasting serum glucose ≥11 mmol/l or use of glucose-lowering drugs. After applying these criteria, 15,248 individuals were available. We further removed ECGs with disturbances that resulted in QRS-detection errors or potentially affect accurate measurement of RR intervals, such as excessive noise, excessive baseline wander, sudden baseline jumps, or spikes. We also removed ECGs with premature ventricular beats, premature supraventricular beats, and second or third degree atrioventricular block. This resulted in 13,943 participants with a low-noise ECG containing only normal beats available for analysis.

This study was approved by the Medical Ethics Committee of the Erasmus University Medical Center. Since all data were anonymized and retrospectively collected, informed consent of the subjects was not required according to Dutch legislation.

### HRV Measurement and Correction

RR intervals for all ECGs were automatically determined by the Modular ECG Analysis System (MEANS), an ECG computer program that has been evaluated extensively (van Bemmel et al., 1990; Willems et al., 1991). The QRS detector of MEANS operates on multiple simultaneously recorded leads. The simultaneous leads are transformed to a detection function, which brings out the QRS complexes among the other parts of the signal. The detection signal is gauged against an adaptable threshold to detect the occurrence of a QRS complex. MEANS signals all the abovementioned signal disturbances, and recognizes premature ventricular complexes, supraventricular complexes, and atrioventricular blocks. All ECGs were visually inspected to validate the automatic processing results. We calculated two time-domain HRV markers: SDNN and RMSSD.

To correct the HRV markers for heart rate (HR), we investigated and compared four different models:

(1) $$\begin{array}{l} Linear:HRV=\alpha +\beta HR\\ Hyperbolic:HRV=\alpha +\beta /HR\\ Parabolic:HRV=\alpha +HR^{\beta }\\ Exponential:HRV=e^{\alpha +\beta HR} \end{array}$$

From these, the following HRV correction formulas can be derived, taking 60 beats per minute as the reference:

(2) $$\begin{array}{l} Linear:HRVc=HRV+\beta (60-HR)\\ Hyperbolic:HRVc=HRV+(1/60-1/HR)\\ Parabolic:HRVc=HRV{\left(60/HR\right)}^{\beta }\\ Exponential:HRVc=HRV^{e\beta (60-HR)} \end{array}$$

To determine the correction parameter β, we used linear regression to fit each of the models (the parabolic and exponential models were log-transformed prior to the regression analysis). To deal with possible confounding by age and sex, regression was performed in predetermined age groups as specified in **Table 1**, and for men and women separately. For each correction model, we determined the *R*-squared value as a measure of model fit for each of the 17 age groups, for SDNN and RMSSD and for men and women separately (17 × 4 = 68 combinations). A mean *R*-squared per model was computed by weighting the *R*-squared values per age group by the number of subjects in that age group.

**Table 1:** Age and sex distribution of the study population.

Age group	No. of boys/men	No. of girls/women	Total
Younger than 1 month	11	8	19
1 to 2 months	27	23	50
3 to 5 months	34	38	72
6 to 11 months	69	54	123
1 to 2 years	51	52	103
3 to 4 years	60	62	122
5 to 7 years	120	104	224
8 to 11 years	115	164	279
12 to 15 years	140	108	248
16 to 19 years	156	382	538
20 to 29 years	450	908	1,358
30 to 39 years	1,376	1,969	3,345
40 to 49 years	962	1,112	2,074
50 to 59 years	976	1,245	2,221
60 to 69 years	995	1,243	2,238
70 to 79 years	295	472	767
80 to 89 years	51	106	157
90 years and older	1	4	5
Total	5,889	8,054	13,943

### Estimation of Normal Values

Centile curves were estimated using the Box-Cox *t* distribution in a semi-parametric model for location, scale and shape (Rigby and Stasinopoulos, 2005; Rigby and Stasinopoulos, 2006). The Box-Cox *t* distribution allows for modeling of the distribution of the median, skewness, and kurtosis as functions of age. The *lms* function of the R-package *gamlss* was used for the creation of the centile curves. The 2nd percentile was taken as the lower limit of normal (LLN) and the 98th percentile as the upper limit of normal (ULN). Normal values for all age categories were estimated using the *predict.gamlss* function of the *gamlss* package. The normal values of all age categories in **Tables 3**, **4** were estimated based on the modeled Box-Cox *t* distribution, taking the central age in the age group. For example, normal values for the category of 16 to 20 years were based on the values of participants aged 18 years.

Differences in LLN, median, and ULN between men and women were tested per each age group, using nonparametric estimates and a bootstrap approach with 5,000 bootstrap samples (Johnson and Romer, 2016).

## Results

**Table 1** shows the age and sex distribution of the study population. All age groups contain more than 100 ECGs, except groups below 6 months and the very sparsely populated group of 90 years or older. Overall, 42% of the subjects are men, varying between 31 and 51% in the individual cohorts (Supplementary Table S1). The age distributions of the cohorts partially overlap (Supplementary Figure S1). Within age groups, the distributions of the HRV markers in the overlapping cohorts are comparable, with small but statistically significant differences between the Leiden and PREVEND cohorts on the one hand, and the Rotterdam and Utrecht cohorts on the other (Supplementary Figures S2, S3).

For each of the four correction models, we determined the *R*-squared values for the 68 combinations of age group, gender, and HRV marker. The exponential model had the highest *R*-squared value for 40 combinations, the parabolic model for 17, the hyperbolic for 9, and the linear model for 2 combinations. The combinations with the highest *R*-squared values of the hyperbolic and linear models were obtained in the four age groups of children less than 1 year, whereas the highest *R*-squared values for the parabolic model were dispersed over all age groups. When the exponential model did not yield the highest *R*-squared, differences with the best model were small (mean ± SD *R*-squared parabolic-exponential 0.008 ± 0.009, hyperbolic-exponential 0.032 ± 0.025, linear-exponential 0.009 ± 0.002). A mean *R*-squared per model was computed by weighting the *R*-squared values per age group by the number of subjects in that age group. **Table 2** indicates that the exponential model overall had the best fit, although differences with the parabolic model were small. Quantile-quantile plots for each of the age groups showed that the residuals for the exponential and parabolic models are roughly normally distributed, contrary to the residuals for the linear and hyperbolic models (Supplementary Figures S4–S7). We therefore decided to use the exponential model for correction of the HRV markers in all age groups. While the estimated parameters α considerably differed across age groups, the parameters β were largely similar (Supplementary Figures S8, S9). We computed an aggregate β as the weighted mean of the age-specific estimates, taking the inverse of the variance of the estimates as the weights (Becker and Wu, 2007). This resulted in the following correction formulas:

**Table 2:** Weighted average R squared for different models that estimate HRV as a function of heart rate.

		Model			
Marker	Sex	Linear	Hyperbolic	Parabolic	Exponential
SDNN	Men	0.098	0.100	0.135	0.138
	Women	0.137	0.145	0.186	0.190
RMSSD	Men	0.158	0.170	0.267	0.270
	Women	0.199	0.217	0.319	0.320

*SDNN, standard deviation of the normal-to-normal RR intervals; RMSSD, root mean square of successive RR-interval differences.*

(3) $$\text{SDNNc }={\text{ SDNN e}}^{-0.02263\;(60-HR)}$$

(4) $$\text{RMSSDc }={\text{ RMSSD e}}^{-0.03243\;(60-HR)}$$

**FIGURE 1:**
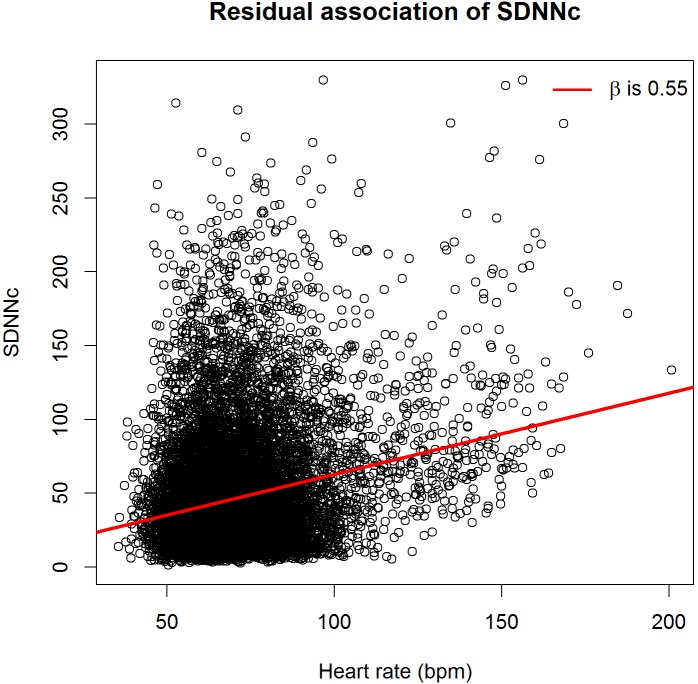
Scatterplot of heart-rate corrected standard deviation of the normal-to-normal RR intervals (SDNNc) versus heart rate.

**FIGURE 2:**
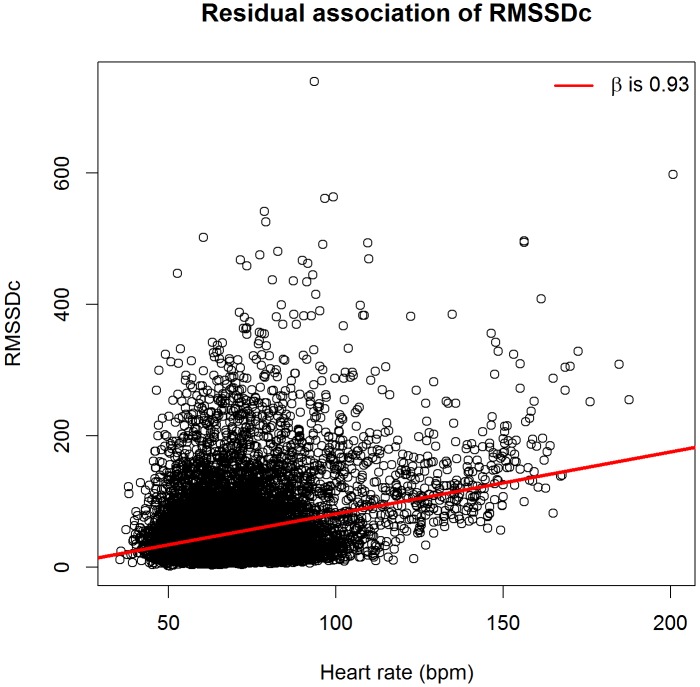
Scatterplot of heart-rate corrected root mean square of successive RR-interval differences (RMSSDc) versus heart rate.

**Figures 1**, **2** show scatterplots of the corrected HRV markers against HR. There was a residual association between corrected HRV markers and HR (linear regression coefficient 0.55 for SDNNc and 0.93 for RMSSDc). If only subjects with HR < 120 beats per minute were considered, the coefficients dropped to 0.37 for SDNNc and 0.61 for RMSSDc. The residual association per age group was generally smaller [median (inter-quartile range) regression coefficients -0.03 (-0.35; 0.05) for SDNNc, and -0.29 (-0.59; 0.70) for RMSSDc]. The highest positive residual associations were found for the age groups of children less than 1 year; the highest negative residual associations occurred for the age groups of children of 5–8 years and for the oldest age group.

We further investigated the effect of using an aggregate β in the correction formula as compared to age-group specific β’s. By definition, the age-group specific β’s minimize the difference between the estimated and observed HRV markers in each separate age group, and there is no residual association between the corrected HRV markers and HR in that age group. However, when we performed age-group specific corrections, the overall residual association only slightly decreased, to 0.47 for SDNNc and 0.64 for RMSSDc.

**FIGURE 3:**
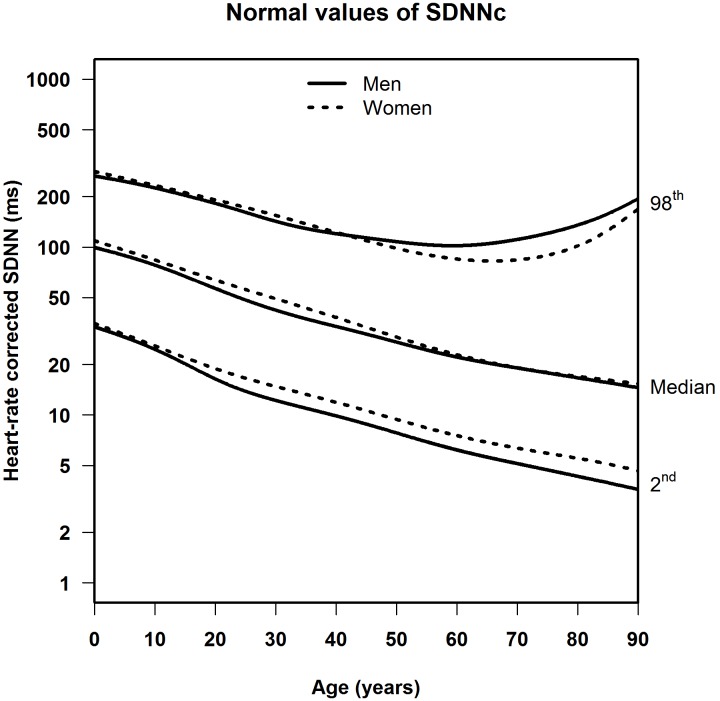
Median, 2nd and 98th percentiles for heart-rate corrected SDNN in men and women.

**FIGURE 4:**
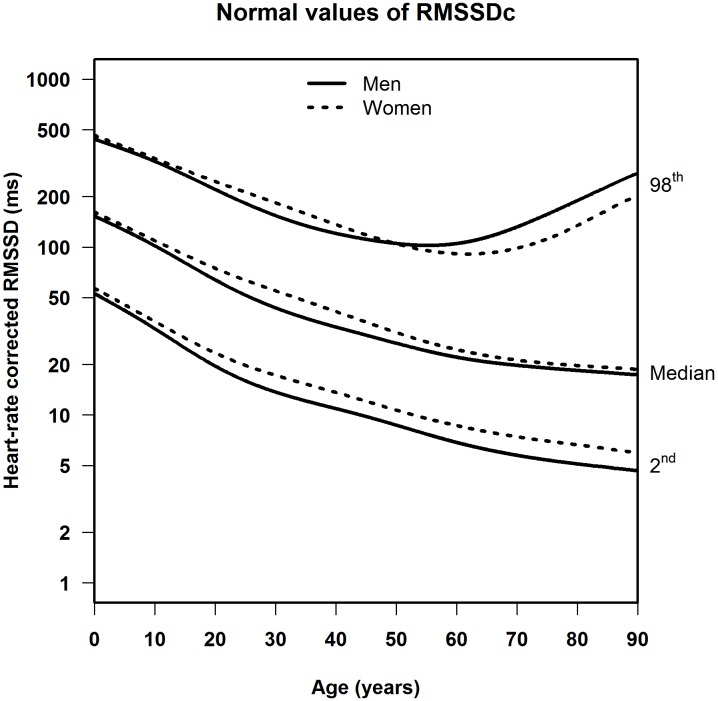
Median, 2nd and 98th percentiles for heart-rate corrected RMSSD in men and women.

The median, LLN and ULN of the heart-rate corrected HRV markers, stratified by sex, are shown in **Table 3** (SDNNc) and **Table 4** (RMSSDc) per age group, and in **Figure 3** (SDNNc) and **Figure 4** (RMSSDc) as continuous age-dependent curves. For comparison, non-parametric percentile estimates of the HRV markers grouped by age decade are shown in Supplementary Figures S10, S11. Other percentile values of SDNNc and RMSSDc are provided as Supplementary Tables S2–S5. SDNNc and RMSSDc display the same age-dependent pattern. The median and LLN of both markers steadily decrease from childhood to the years of middle and older age. The ULN also decreases till the age of 50–60, after which both markers show a marked increase of their ULN, resulting in a greater range of normal values in the elderly. The differences between men and women appear to be small, but are statistically significant mostly for the LLN and median in the age groups of 20–70 years (see **Tables 3**, **4**), with lower values for men than women. The differences in the ULN between men and women are generally not statistically significant.

**Table 3:** Normal values for heart-rate corrected SDNN (in ms) per age group and for both sexes.

	Median (2nd percentile; 98th percentile)	
Age group	Boys/men	Girls/women
<1 month	99.6 (33.6; 265.6)	109.2 (35.1; 282.2)
1 to 2 months	99.4 (33.4; 265.1)	108.8 (35.0; 281.6*)
3 to 5 months	98.8 (33.2; 264.1)	108.1 (34.7; 280.3)
6 to 11 months	98.1 (32.9; 262.7)	107.1 (34.3; 278.3)
1 to 2 years	95.4 (31.8; 258.0)	103.8 (33.1; 271.9)
3 to 4 years	91.3 (30.0; 250.4)	98.6 (31.2; 261.9)
5 to 7 years	86.0 (27.8; 240.5)	92.3 (28.9; 249.8)
8 to 11 years	78.3 (24.7; 225.7)	84.0 (25.8; 233.5)
12 to 15 years	69.3 (21.1; 208.0)	75.2 (22.7; 215.7)
16 to 19 years	60.7 (17.8; 190.9)	67.3 (20.0; 199.2)
20 to 29 years	48.5 (13.9; 161.4)	56.0*** (16.6**; 172.7)
30 to 39 years	37.5 (11.0; 129.2)	43.4*** (13.3**; 137.8)
40 to 49 years	30.4 (8.8; 113.7)	33.3* (10.6; 109.5)
50 to 59 years	24.4 (6.9; 103.4)	25.6 (8.4***; 90.2*)
60 to 69 years	20.4 (5.6; 104.8)	20.7 (6.9**; 82.8)
70 to 79 years	17.8 (4.7; 120.9)	17.9 (5.9; 89.5*)
80 to 89 years	15.6 (3.9; 158.3)	16.1 (5.1; 126.1)

*^∗^P-value < 0.05 for difference between men and women; ^∗∗^P-value < 0.01; ^∗∗∗^P-value < 0.001; SDNN, standard deviation of the normal-to-normal RR intervals.*

**Table 4:** Normal values for heart-rate corrected RMSSD (in ms) per age group for both sexes.

	Median (2nd percentile; 98th percentile)	
Age group	Boys/men	Girls/women
<1 month	153.1 (53.0; 440.2)	161.9 (56.9; 463.9)
1 to 2 months	152.4 (52.7; 438.7)	161.1 (56.6; 462.2)
3 to 5 months	150.9 (52.1; 435.7)	159.6* (56.0; 458.6)
6 to 11 months	148.8 (51.2; 431.1)	157.3* (55.1; 453.2)
1 to 2 years	141.9 (48.4; 416.3)	150.0 (52.1; 435.8)
3 to 4 years	131.4 (44.1; 393.1)	138.9 (47.6; 409.4)
5 to 7 years	118.8 (39.1; 364.6)	126.0 (42.5; 378.3)
8 to 11 years	102.1 (32.8; 324.9)	109.7 (36.1; 338.1)
12 to 15 years	84.8 (26.5; 280.3)	93.6 (30.1; 297.1)
16 to 19 years	70.1 (21.6; 239.3)	80.4 (25.3; 261.8)
20 to 29 years	51.9 (16.0; 182.7)	63.7*** (19.8**; 212.9)
30 to 39 years	37.7 (12.1; 134.4)	47.7*** (15.3*; 158.4)
40 to 49 years	29.9 (9.8; 111.5)	35.8*** (12.1; 118.5)
50 to 59 years	24.1 (7.7; 102.5)	27.3*** (9.5***; 95.6)
60 to 69 years	20.7 (6.2; 114.6)	22.6** (8.0*; 92.2)
70 to 79 years	19.0 (5.4; 157.1)	20.3 (7.0; 112.1*)
80 to 89 years	17.9 (4.9; 230.1)	19.2 (6.3; 166.7)

*^∗^P-value < 0.05 for difference between men and women; ^∗∗^P-value < 0.01; ^∗∗∗^P-value < 0.001; RMSSD, root mean square of successive RR-interval differences.*

**FIGURE 5:**
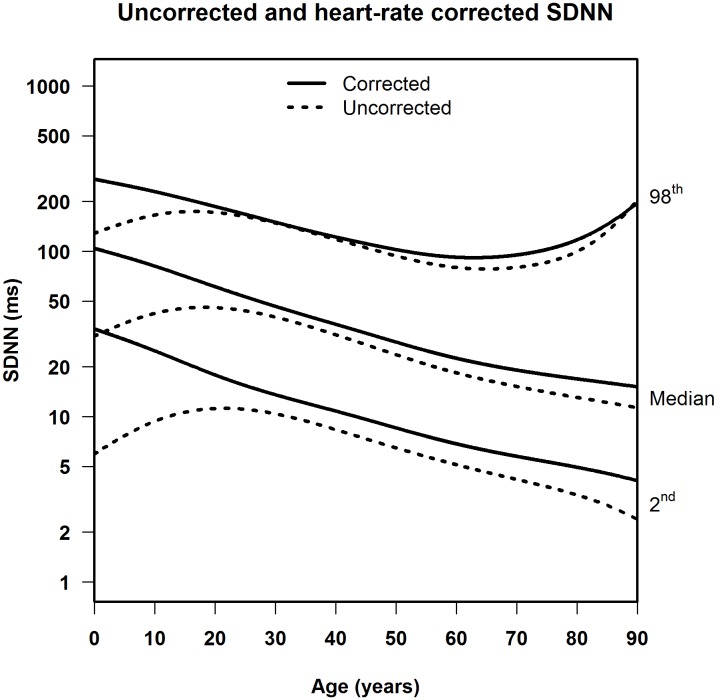
Median, 2nd and 98th percentiles for heart-rate corrected SDNN and uncorrected SDNN.

**FIGURE 6:**
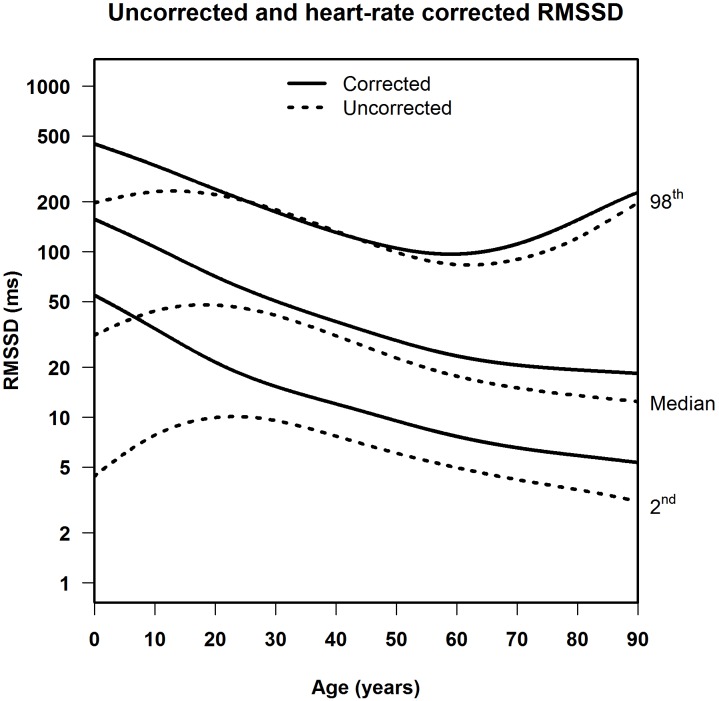
Median, 2nd and 98th percentiles for heart-rate corrected RMSSD and uncorrected RMSSD.

**Figures 5**, **6** illustrate the effect of heart-rate correction on the normal values of SDNN and RMSSD, respectively. The marked difference between the corrected and uncorrected HRV markers in infancy and adolescence can be explained by the strong age-dependence of heart rate in children (Supplementary Figure S12). The lower the age, the higher the average heart rate, and thus the larger the difference between corrected and uncorrected HRV markers. Since average heart rates in middle-aged and elderly people are fairly constant (65–70 beats per minute, see Supplementary Figure S12), the correction factor in the correction formulas is also fairly constant (e.g., for SDNN 1.12 for 65 bpm and 1.25 for 70 bpm). As a result, the corrected values for these age groups, on average, differ from the uncorrected by a small, constant factor.

## Discussion

This is the first population-based study to provide heart-rate corrected normal values for SDNN and RMSSD as derived from 10-s ECGs across all ages and for both sexes. Our study shows that in both men and women, the LLN of SDNNc and RMSSDc decreases continuously from birth to old age, whereas the ULN decreases at the same rate until the age of 50–60 and then starts to rise again. The differences in SDNNc and RMSSDc between men and women are small, with men generally having slightly lower LLN and median values than women in the age groups of 20–70.

Several studies calculated uncorrected normal values for HRV from 5-min or 24-h ECG signals (Umetani et al., 1998; Nunan et al., 2010; Kim and Woo, 2011; Seppala et al., 2014). Nunan et al. (2010) published normal values for middle-aged and elderly people in a systematic review of 5-min SDNN and RMSSD using 44 studies containing 21,438 participants. However, their data were not stratified by age. Seppala et al. (2014) reported normal HRV values but only for children aged 6–8 years. Additionally, two studies looked at the effect of age. Kim and Woo (2011) found that 5-min SDNN and RMSSD decreased between the age of 18 and 50 in both men and women. Umetani et al. (1998) also found that 24-h SDNN decreases in adults, as recorded in 260 healthy participants aged 10–99 years. The decrease of uncorrected 5-min or 24-h HRV in aging adults was similar to the decrease in uncorrected 10-s HRV found in our study, as illustrated in **Figure 5**. There is one other study that calculated normal values of uncorrected 10-s HRV, for middle-aged and elderly participants, but this study did not take age into account (O’Neal et al., 2016).

When HRV is not heart-rate corrected, we find a sharp increase between birth and adolescence, a pattern that was also observed by others (Silvetti et al., 2001; Karlsson et al., 2012). However, this increase is connected to heart rate, which is strongly age-dependent in the young (Rijnbeek et al., 2001). After heart-rate correction, SDNNc and RMSSDc decrease continuously from birth to adolescence, and further into higher ages. A continuous decrease in HRV indices was also observed in a recent study in children aged 6–13 years (Gasior et al., 2015), after correcting the indices by different powers of the heart rate. These findings underline the fact that meaningful comparison of HRV measurements, and their possible association with adverse outcomes, can only be made if the relationship between HRV and heart rate is properly taken into account. Our correction formulas for SDNN and RMSSD can be applied to deal with this issue.

We compared several types of formulas to correct the HRV markers for heart rate. The exponential correction formula yielded the best model fit for most of the age groups, although differences with a parabolic correction formula were small. For some of the age groups below 1 year, linear or hyperbolic formulas gave a better model fit, but differences with the exponential formula were small. Moreover, quantile–quantile plots showed that the residuals of the exponential model were more normally distributed. We therefore recommend use of the exponential correction formula. It should be noted though that this correction is not perfect as there remained some residual association with heart rate, especially for high heart rates as often seen in children. Use of a separate correction formula per age group turned out to reduce this residual association only slightly. This may be explained by differences in the distribution of the residuals between age groups, which when combined can still result in an overall residual association. For practical reasons, we decided to use a single correction formula for all ages. A correction formula without overall residual association can be obtained by fitting the model on all data together, i.e., without taking age into account, but this model turned out to have a very poor fit (*R*-squared 0.08 for SDNN and 0.15 for RMSSD) and was therefore not further considered.

We made the observation, not previously reported in the literature, that after the age of 60 the ULN turns upwards, in men even more than in women, while the median and LLN continue their downward course. This finding implies a growing instability in sinus node activity in a part of the aging population. This is perhaps caused by incipient dysfunction of the cardiac excitation and conduction system.

Our study has a number of strengths. We are the first study to report HRV normal values that are corrected for heart rate. We have a total of 13,943 ECGs with wide age coverage, from children of 11 days to 90-year old, both male and female. All ECGs from the five included study cohorts were analyzed automatically by a well-validated program, MEANS, which eliminates intra-observer variation that may result from manual measurement of RR intervals. The use of the 10-s ECG may be seen as a strength and as a limitation. Admittedly, the 10-s ECG contains less information than longer recordings, and may sometimes contain only a few RR intervals for HRV calculation. Also, HRV estimates based on 10-s ECGs will have a larger variability than HRV estimates from longer recordings. In that respect, longer recordings are to be preferred over 10-s ECGs. On the other hand, the 10-s ECG is in universal use, cheap, and easily and quickly obtained. For many cohort studies, 5-min or longer ECG signals are not available, and 10-s HRV would be the only option to study HRV in these cohorts. A further limitation of our study is the low number of ECGs in the extremes of the age distribution. For this reason, the normal limits of the groups younger than 6 months and older than 90 years should be used with caution. Another limitation is that the *R*-squared values for the exponential model, while being better than for the other models, are still rather low. Thus, the exponential model is not optimal. Finally, we excluded individuals who used beta blockers, probably the most commonly used drugs that affect heart rate and HRV. Other drugs, such as tricyclic antidepressants, have also been reported to influence HRV (Noordam et al., 2016) but were not excluded because this medication information was not available in our study. However, in previous studies that showed an effect of medication on HRV, the HRV markers were not heart-rate corrected as we propose here, which may explain part of the observed effect.

## Conclusion

Normal limits have been established for heart-rate corrected SDNN and RMSSD, derived from 10-s ECGs, using a consistent and automatic methodology for all ages and both sexes. Our coverage of the pediatric population allows age-specific comparisons of HRV of the pediatric ECGs, from birth to puberty, independent of the rapid change in heart rate in this period of life. Using these normal values, both researchers and clinicians have a tool to decide upon cut-off values of HRV.

## Author Contributions

MvdB, PR, and JK: conception and design of the work. MvdB, PR, MN, AH, MB, HH, CS, ME, BS, and JK: data collection, cleaning, preprocessing. MvdB, PR, GvH, and JK: statistical analysis and interpretation and drafting of the manuscript. All authors: critical review of the manuscript and final approval.

## Conflict of Interest Statement

The authors declare that the research was conducted in the absence of any commercial or financial relationships that could be construed as a potential conflict of interest.
