# Corrigendum: Normal Values of Corrected Heart-Rate Variability in 10-Second Electrocardiograms for All Ages

**DOI:** 10.3389/fphys.2019.01373

**Published:** 2019-11-01

**Authors:** Marten E. van den Berg, Peter R. Rijnbeek, Maartje N. Niemeijer, Albert Hofman, Gerard van Herpen, Michiel L. Bots, Hans Hillege, Cees A. Swenne, Mark Eijgelsheim, Bruno H. Stricker, Jan A. Kors

**Affiliations:** ^1^Department of Medical Informatics, Erasmus University Medical Center, Rotterdam, Netherlands; ^2^Department of Epidemiology, Erasmus University Medical Center, Rotterdam, Netherlands; ^3^Julius Center for Health Sciences and Primary Care, University Medical Center Utrecht, Utrecht, Netherlands; ^4^Department of Cardiology, University Medical Center Groningen, Groningen, Netherlands; ^5^Department of Cardiology, Leiden University Medical Center, Leiden, Netherlands; ^6^Department of Internal Medicine, University Medical Center Groningen, Groningen, Netherlands; ^7^Department of Internal Medicine, Erasmus University Medical Center, Rotterdam, Netherlands; ^8^Health and Youth Care Inspectorate, Utrecht, Netherlands

**Keywords:** electrocardiography, heart-rate variability, normal values, heart-rate correction, children, adults, elderly

In the original article, there was a mistake in Table S5 as published. The normal values for “SDNN” were given instead of for “RMSSD.” The corrected Table S5 appears below.

**Table S5:** Percentiles of heart-rate corrected RMSSD (in milliseconds) for women.

**Age group**	**2^**nd**^**	**5^**th**^**	**10^**th**^**	**25^**th**^**	**50^**th**^**	**75^**th**^**	**90^**th**^**	**95^**th**^**	**98^**th**^**
< 1 month	56.9	70.8	85.5	116.1	161.9	225.9	307.2	371.6	463.9
1 to 3 months^*^	56.6	70.5	85.1	115.5	161.1	224.9	305.9	370.1	462.2
3 to 6 months	56.0	69.7	84.2	114.4	159.6	222.8	303.3	367.0	458.6
6 to 12 months	55.1	68.6	82.9	112.7	157.3	219.8	299.4	362.5	453.2
1 to 3 years	52.1	65.0	78.7	107.2	150.0	210.0	286.7	347.7	435.8
3 to 5 years	47.6	59.7	72.4	98.9	138.9	195.1	267.3	325.2	409.4
5 to 8 years	42.5	53.5	65.1	89.4	126.0	177.8	244.8	298.9	378.3
8 to 12 years	36.1	45.9	56.1	77.5	109.7	155.7	215.8	265.0	338.1
12 to 16 years	30.1	38.5	47.3	65.7	93.6	133.6	186.7	230.7	297.1
16 to 20 years	25.3	32.6	40.3	56.2	80.4	115.3	162.1	201.5	261.8
20 to 30 years	19.8	25.6	31.7	44.5	63.7	91.6	129.5	162.0	212.9
30 to 40 years	15.3	19.7	24.2	33.6	47.7	68.2	96.2	120.3	158.4
40 to 50 years	12.1	15.3	18.6	25.4	35.8	50.8	71.5	89.6	118.5
50 to 60 years	9.5	11.9	14.4	19.5	27.3	38.9	55.5	70.5	95.6
60 to 70 years	8.0	9.9	11.9	16.1	22.6	32.7	48.2	63.6	92.2
70 to 80 years	7.0	8.8	10.6	14.4	20.3	30.2	47.2	66.9	112.1
80 to 90 years	6.3	8.1	9.8	13.5	19.2	29.3	49.7	78.4	166.7

* *The term “to” specifies the upper limit in the sense of “less than”*.

The authors apologize for this error and state that this does not change the scientific conclusions of the article in any way. The original article has been updated.

