# Supplementary material for: Normal Values of Corrected Heart-Rate Variability in 10-Second Electrocardiograms for All Ages
Source: Front Physiol. 2018 Apr 27;9:424. doi: 10.3389/fphys.2018.00424 (PMC5934689; doi:10.3389/fphys.2018.00424)
Supplement: Supplementary file 1 [file Data_Sheet_1.PDF]

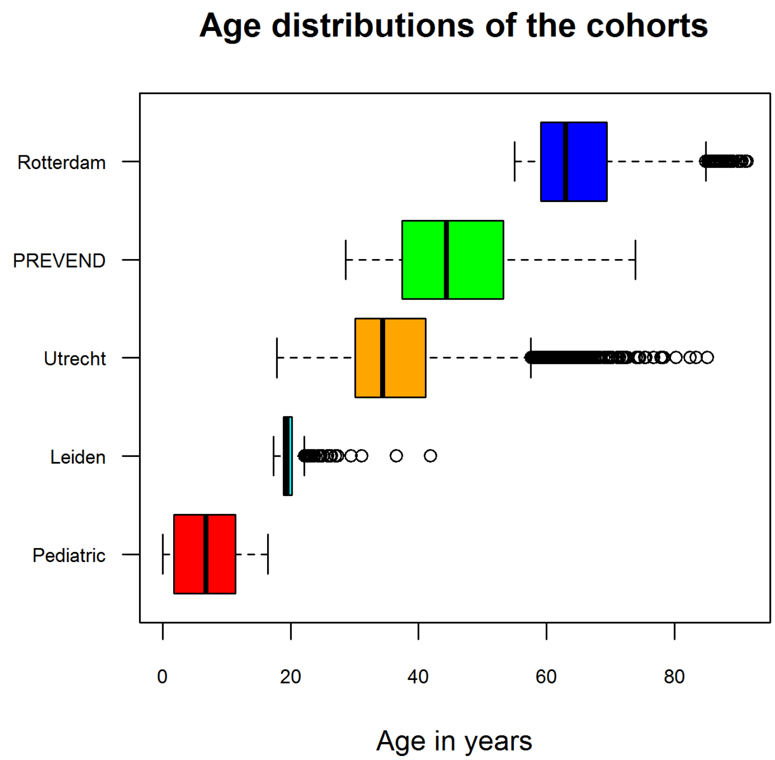

Figure S1. Age distributions of the different cohorts.

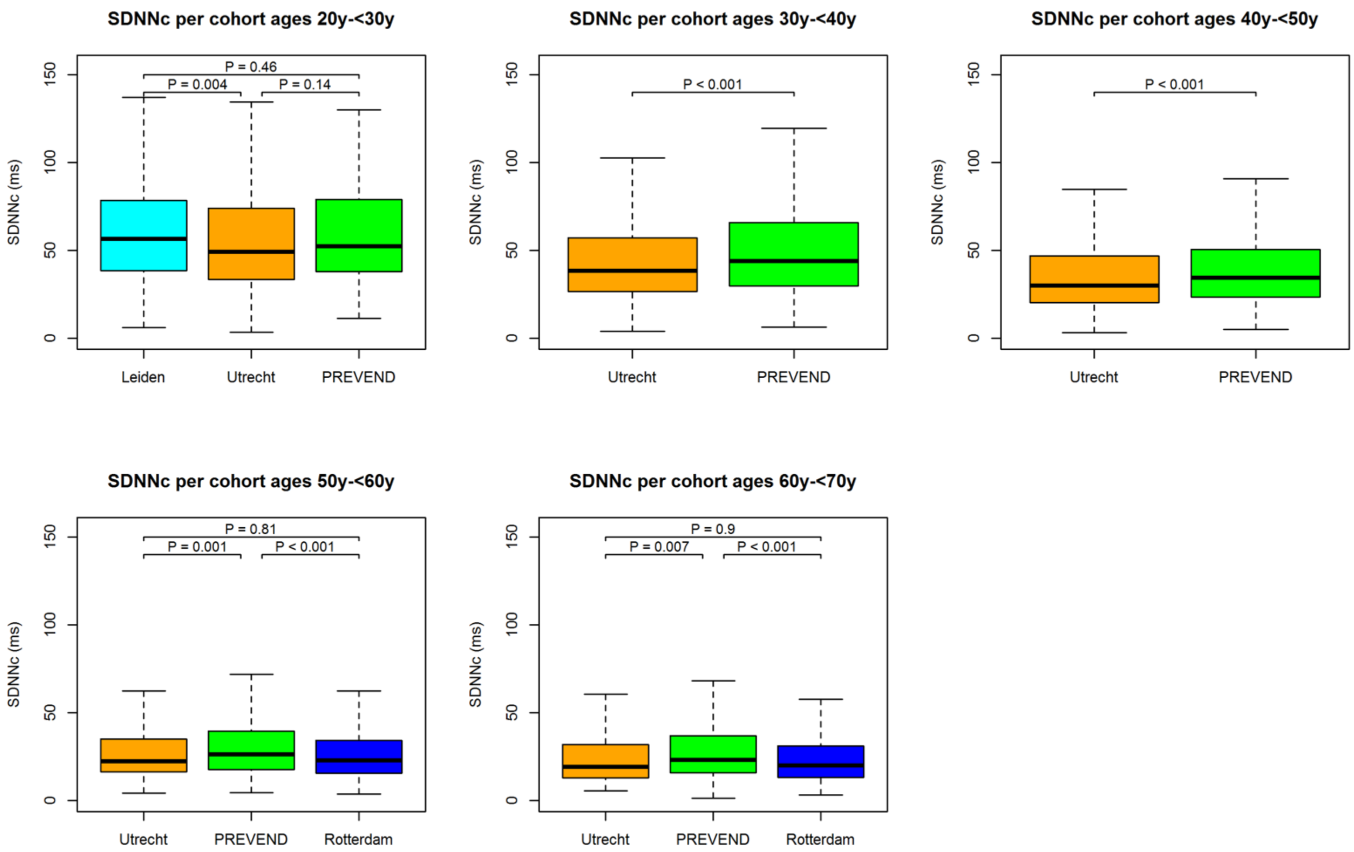

Figure S2. Distribution of SDNNc per age group for cohorts with overlapping age distributions. For each age group, only the cohorts with more than 100 subjects in that age group are shown. Differences between cohorts were tested with the Wilcoxon rank-sum test.

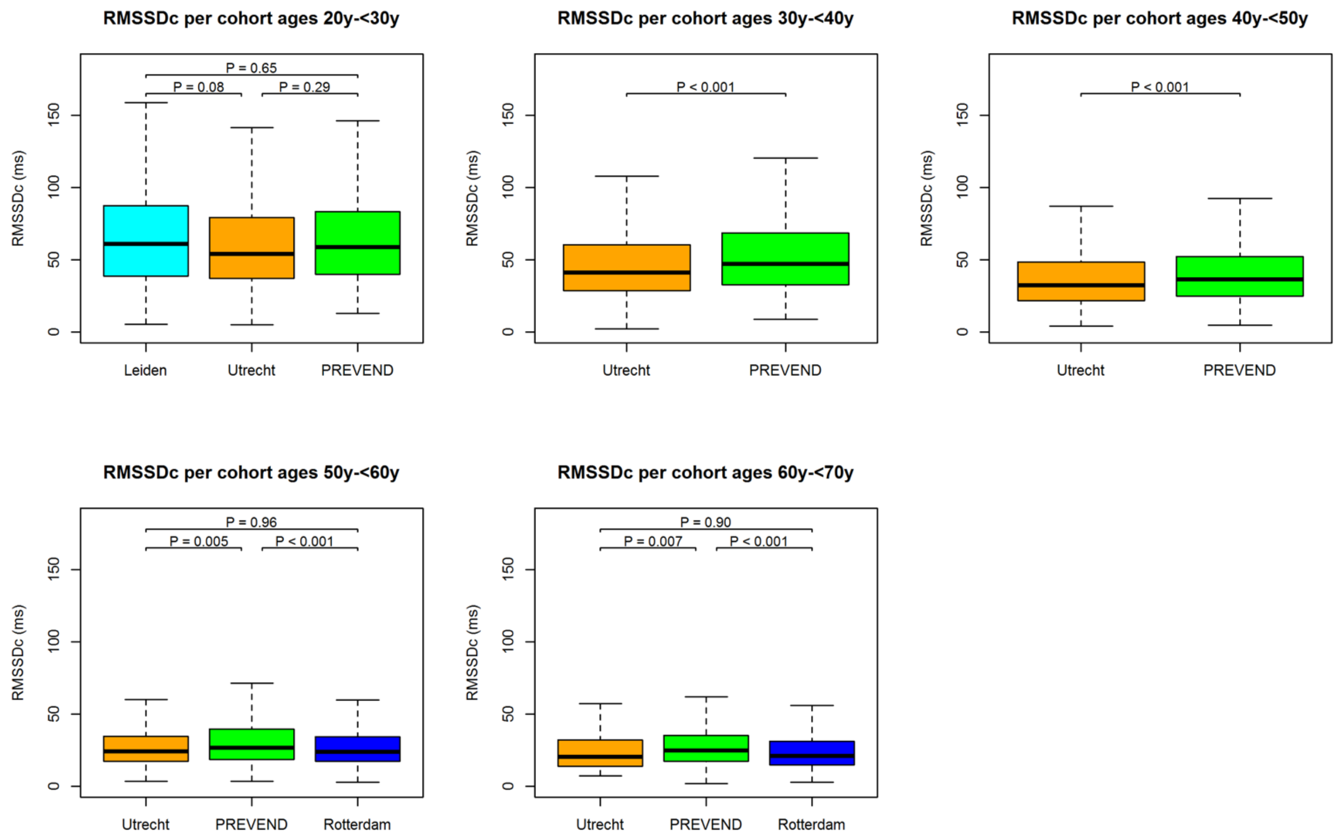

Figure S3. Distribution of RMSSDc per age group for cohorts with overlapping age distributions. For each age group, only the cohorts with more than 100 subjects in that age group are shown. Differences between cohorts were tested with the Wilcoxon rank-sum test.

Quantile-Quantile plots for the linear model - SDNN

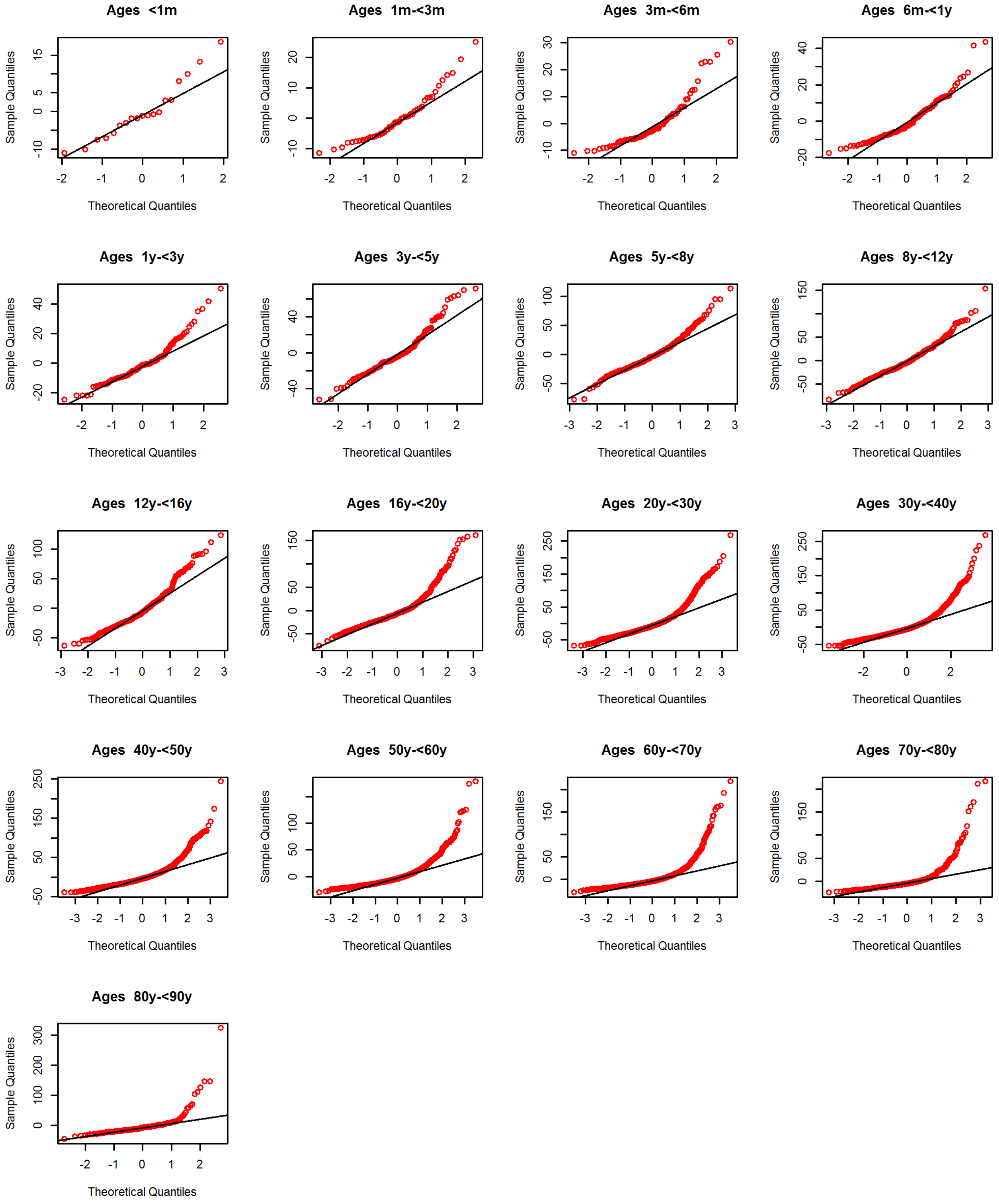

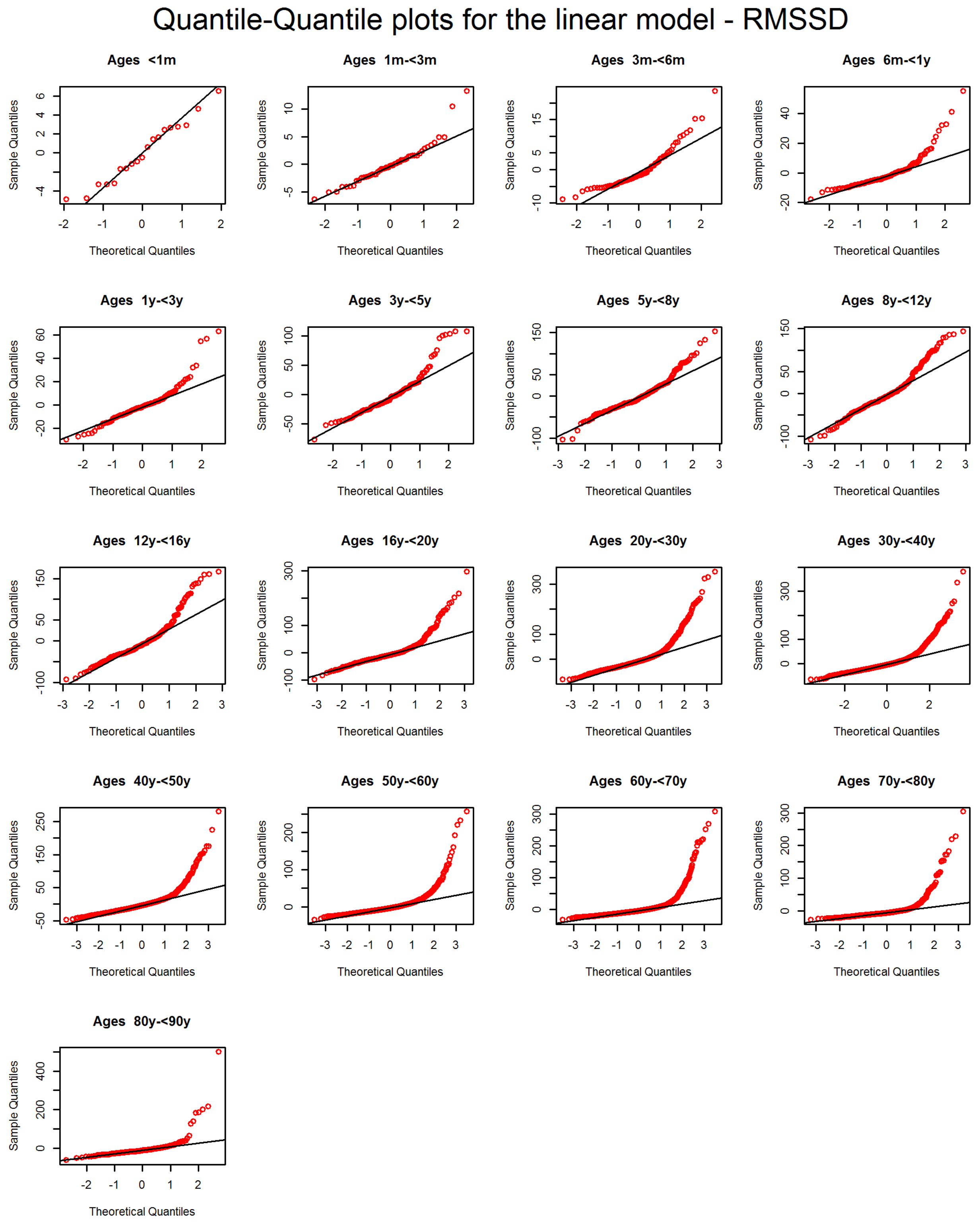

Figure S4. Quantile-quantile plots for the linear model per age group.

Quantile-Quantile plots for the hyperbolic model - SDNN

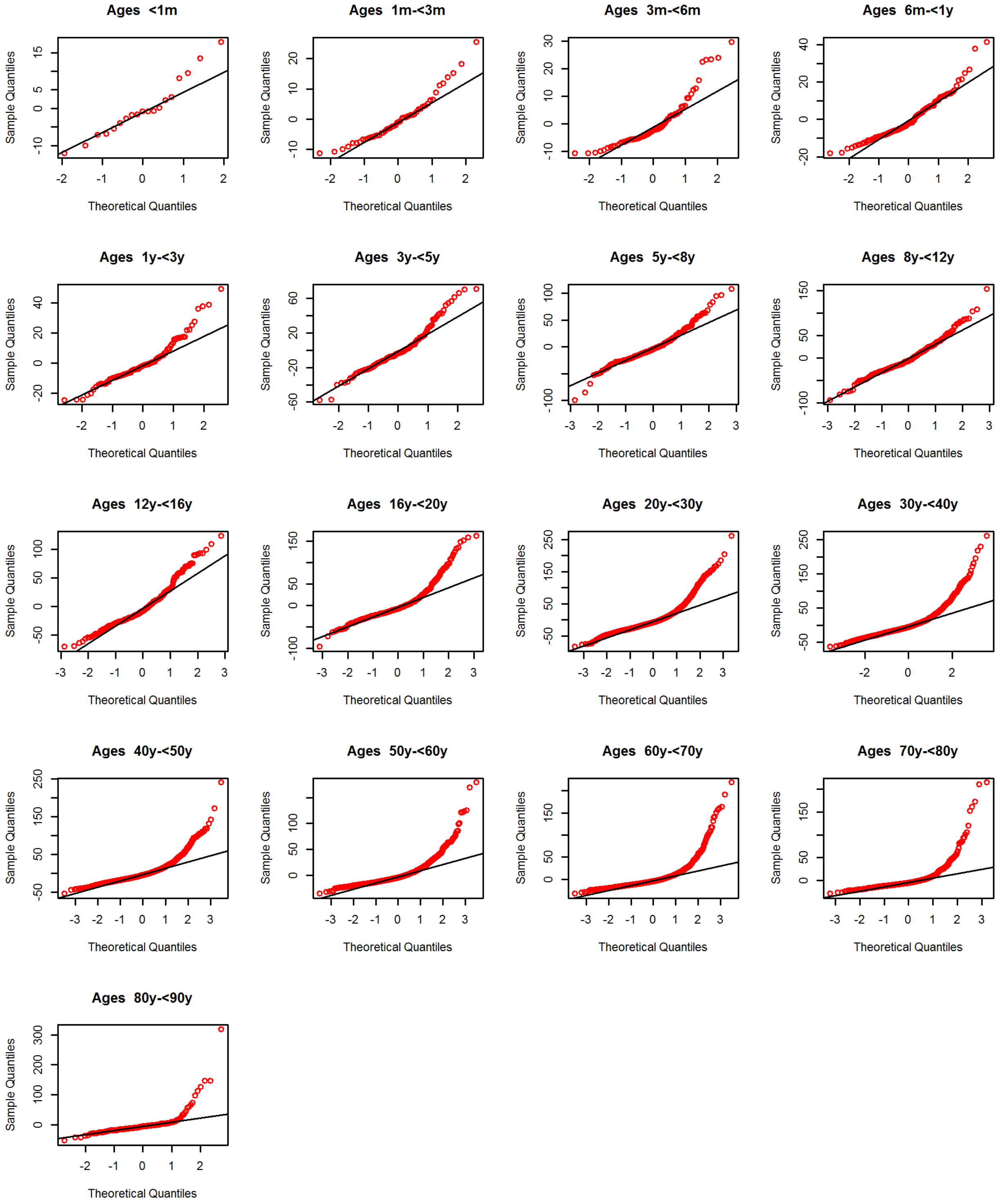

Quantile-Quantile plots for the hyperbolic model - RMSSD

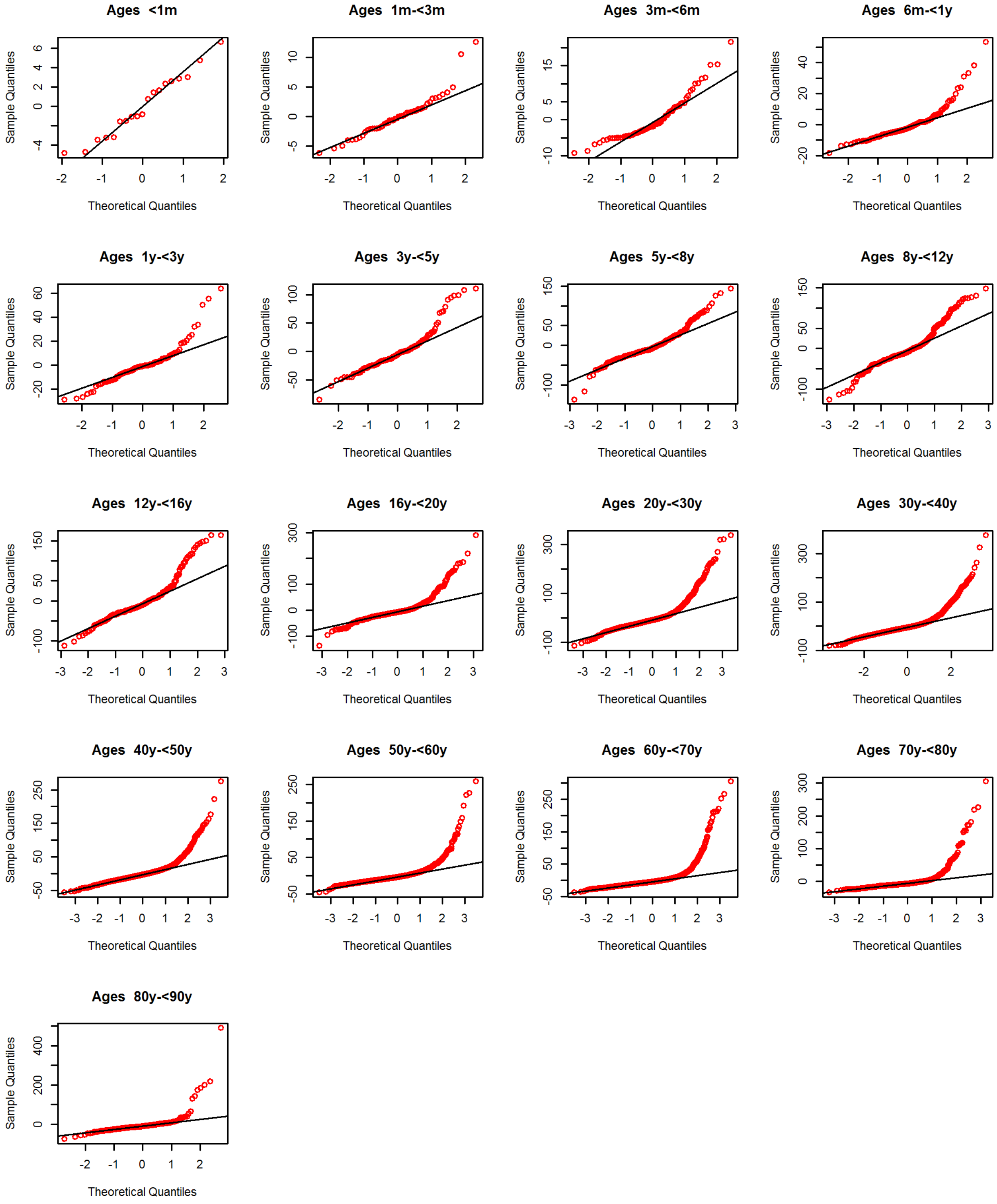

Figure S5. Quantile-quantile plots for the hyperbolic model per age group.

Quantile-Quantile plots for the parabolic model - SDNN

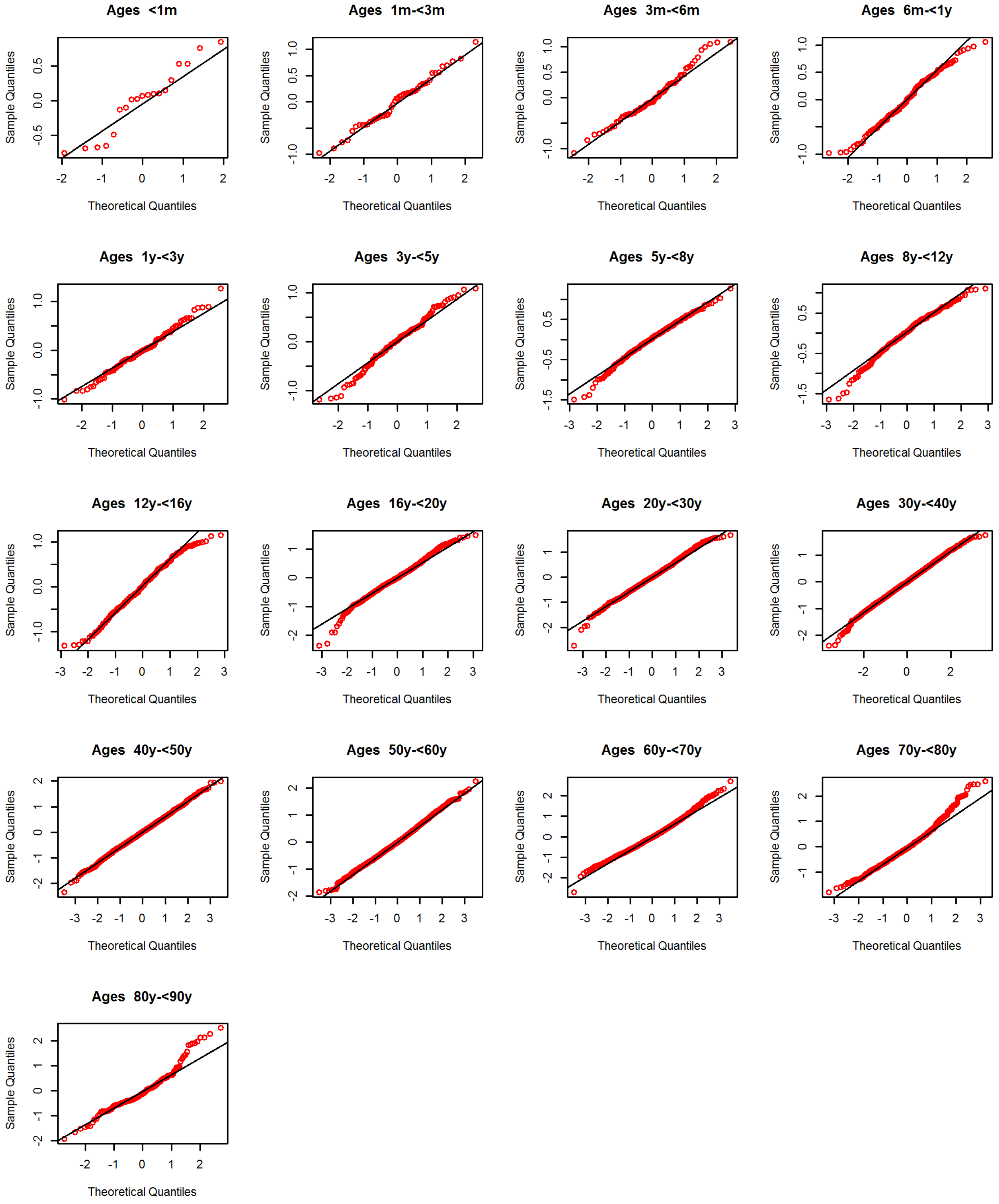

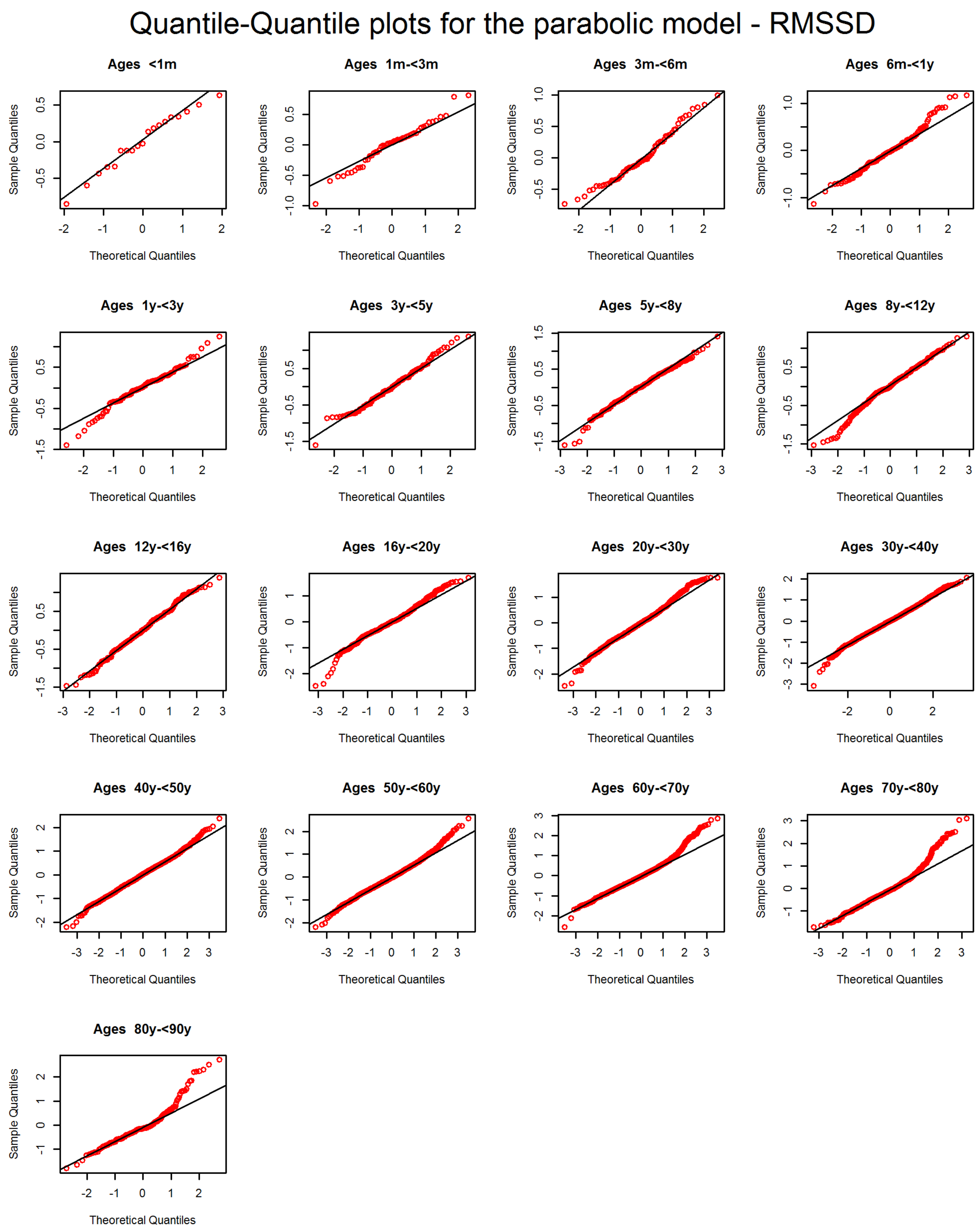

Figure S6. Quantile-quantile plots for the parabolic model per age group.

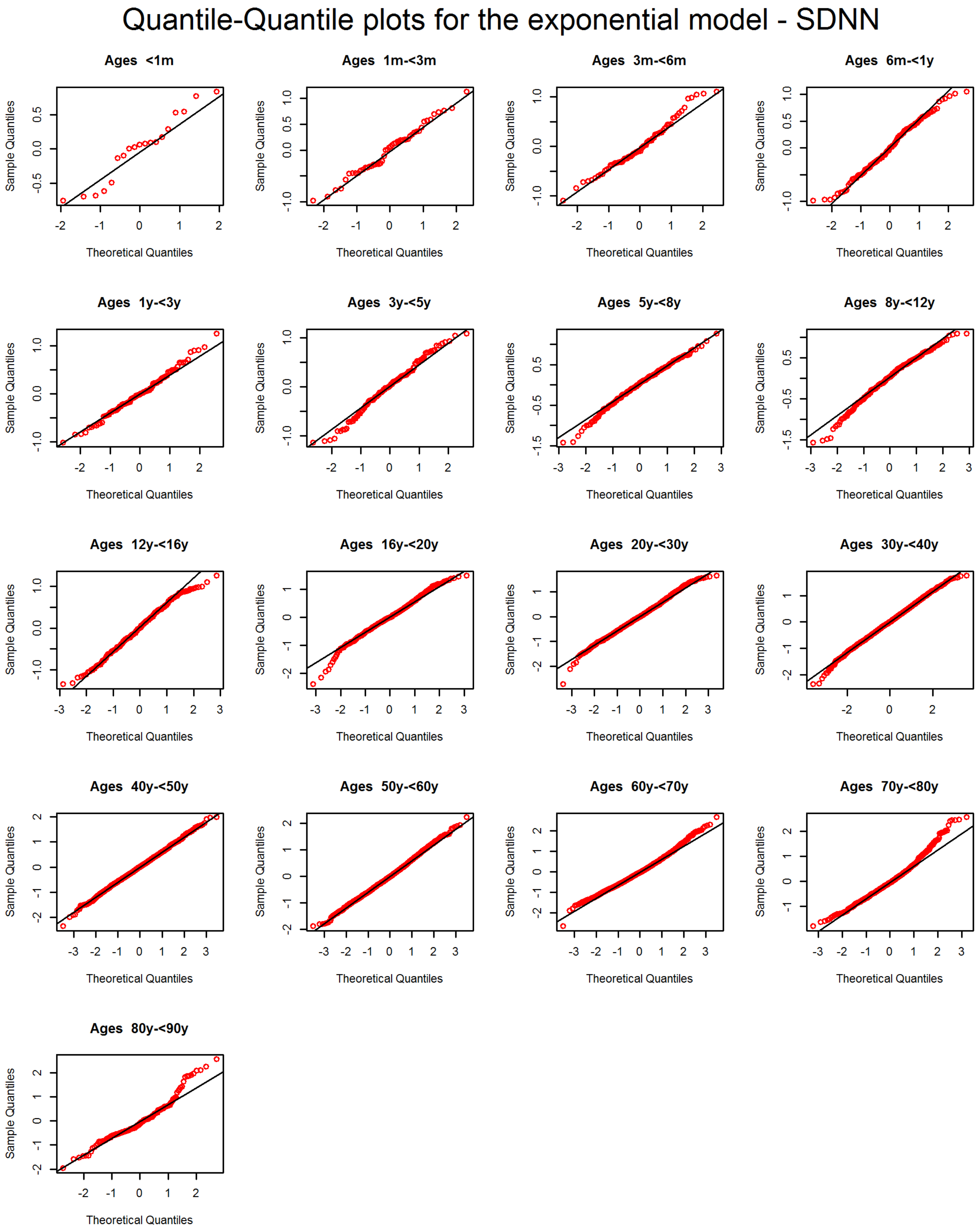
Quantile-Quantile plots for the exponential model - SDNN
Ages <1m
Ages 1m-<3m
Ages 3m-<6m
Ages 6m-<1y
Ages 1y-<3y
Ages 3y-<5y
Ages 5y-<8y
Ages 8y-<12y
Ages 12y-<16y
Ages 16y-<20y
Ages 20y-<30y
Ages 30y-<40y
Ages 40y-<50y
Ages 50y-<60y
Ages 60y-<70y
Ages 70y-<80y
Ages 80y-<90y
Sample Quantiles
Theoretical Quantiles

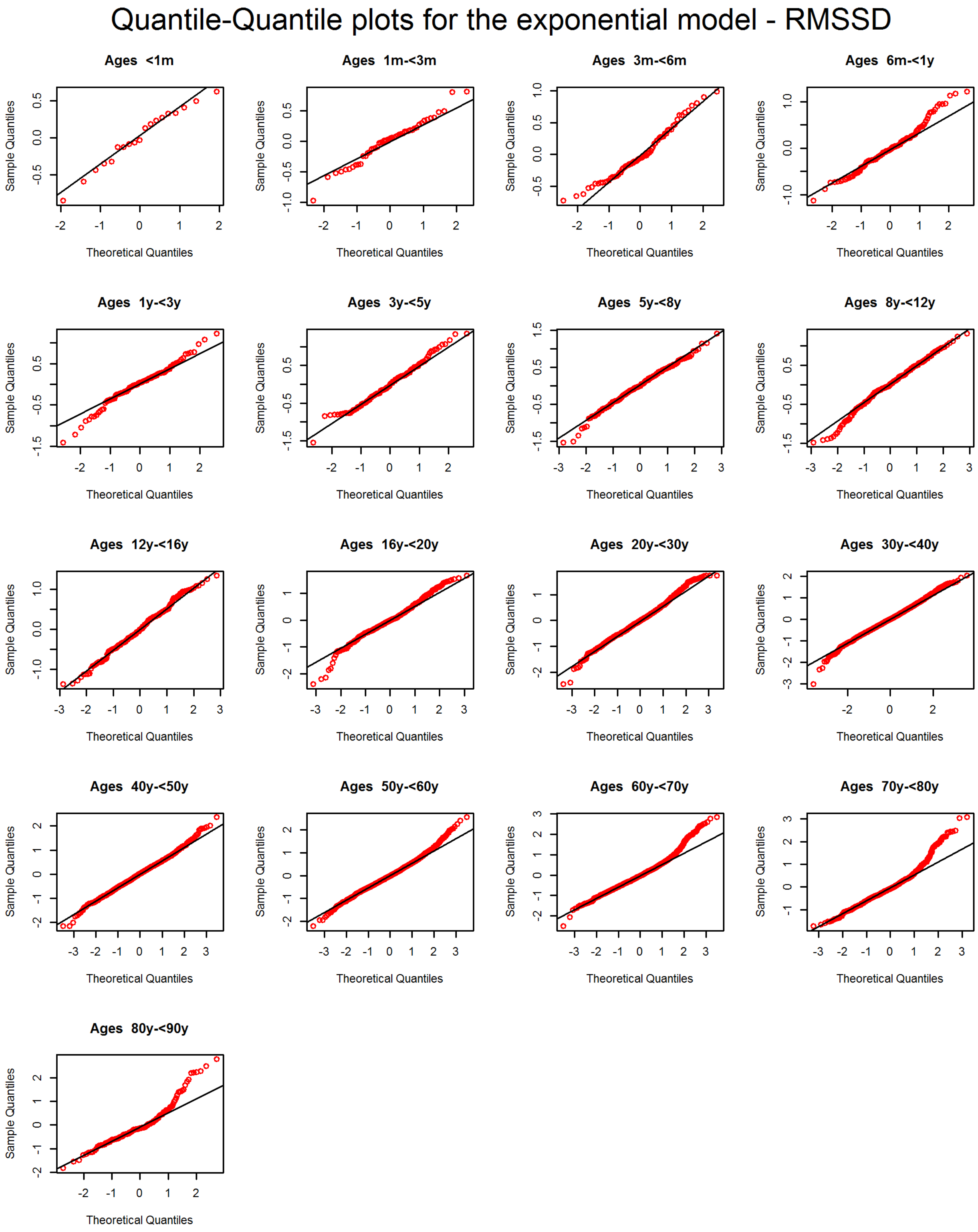

Figure S7. Quantile-quantile plots for the exponential model per age group.

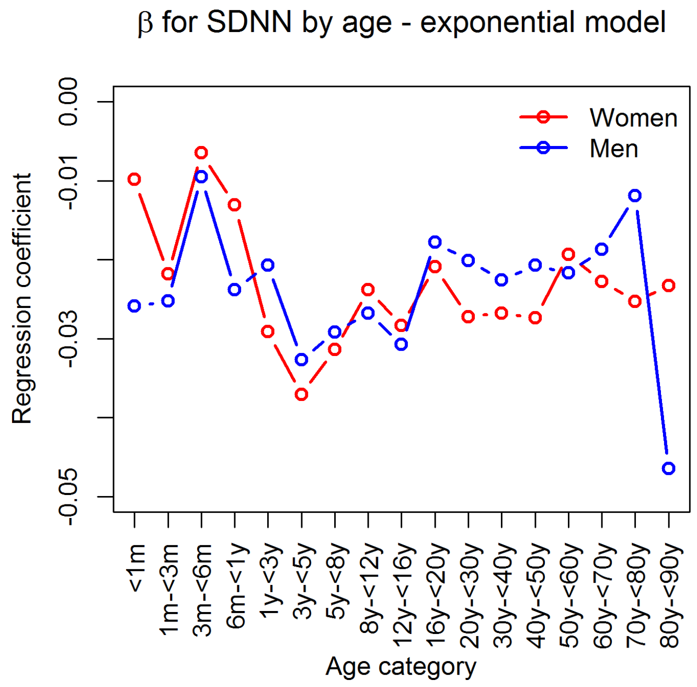

Figure S8. Regression coefficients of the exponential model for SDNN per age group and sex.

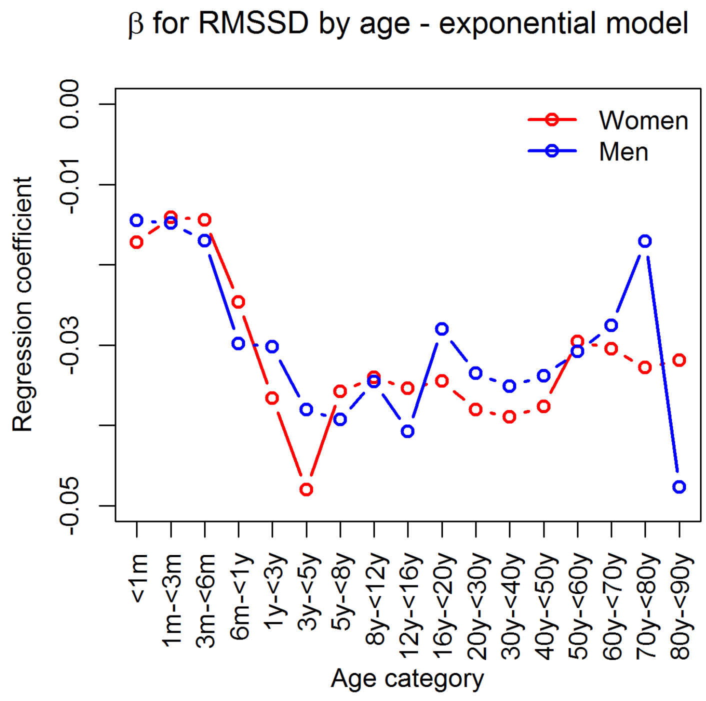

Figure S9. Regression coefficients of the exponential model for RMSSD per age group and sex.

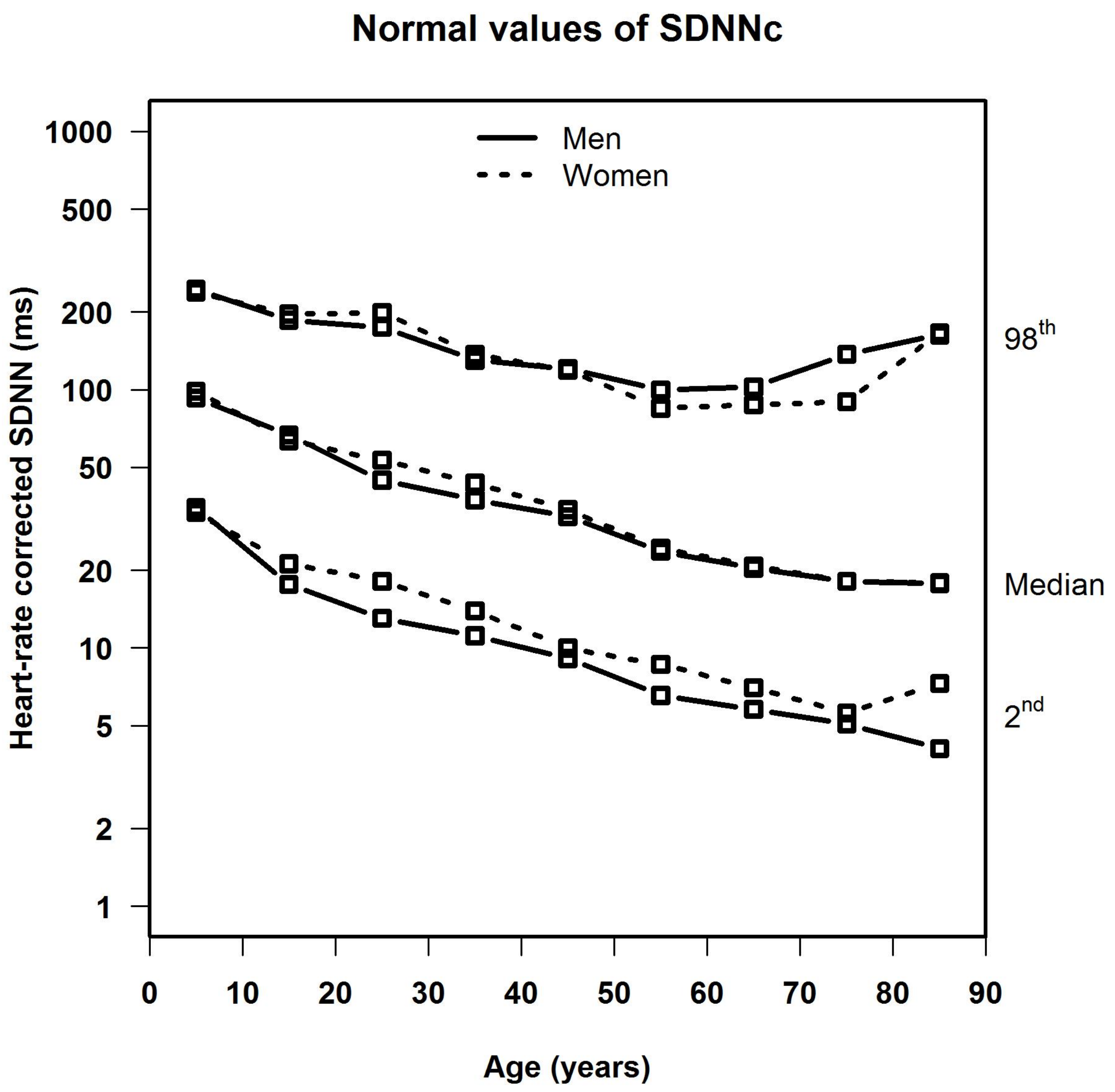

Figure S10. Median, 2nd and 98th percentiles for heart-rate corrected SDNN in men and women grouped by age decade.

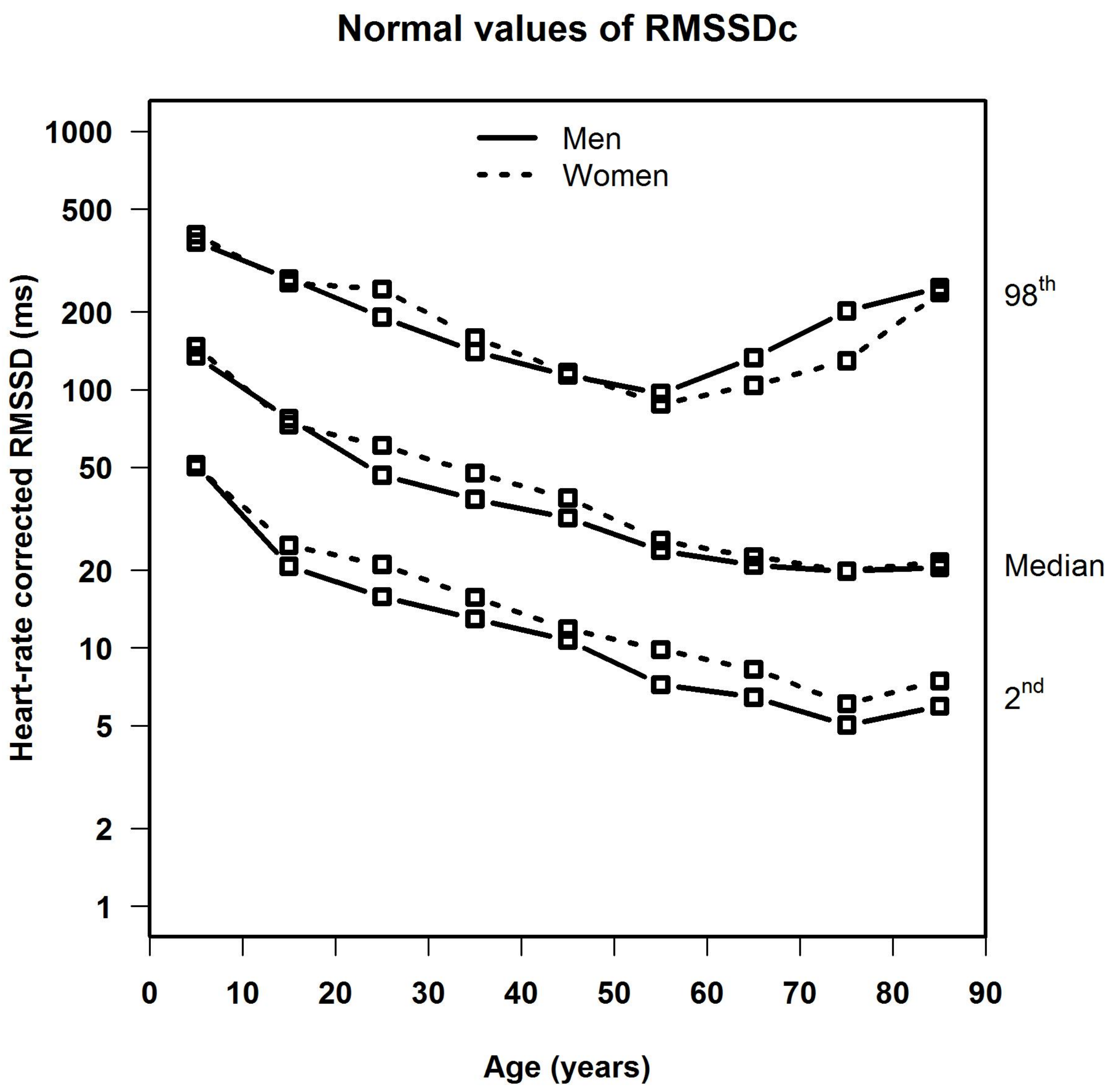

Figure S11. Median, 2nd and 98th percentiles for heart-rate corrected SDNN in men and women grouped by age decade.

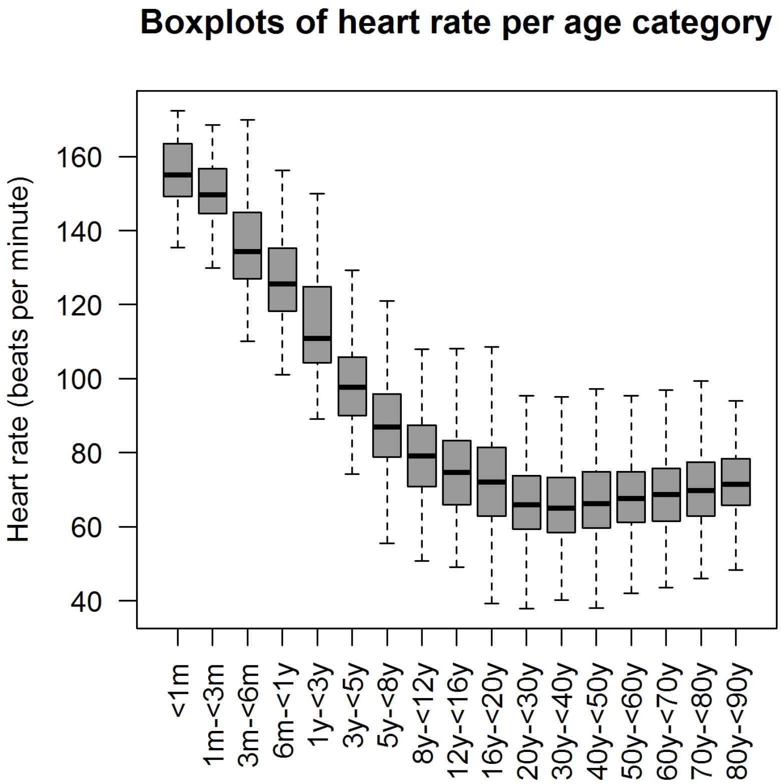

Figure S12. Heart-rate distribution per age group.

Table S1. Sex distribution for the different cohorts.

Cohort	Men	Women
Pediatric Normal ECG Study	640 (51%)	618 (49%)
Leiden University Project	224 (31%)	494 (69%)
Utrecht Health Project	1,697 (41%)	2,400 (59%)
PREVEND Study	1,961 (44%)	2,511 (56%)
Rotterdam Study	1,367 (40%)	2,031 (60%)
All	5,889 (42%)	8,054 (58%)

Table S2. Percentiles of heart-rate corrected SDNN (in milliseconds) for men.

Age group	2nd	5th	10th	25th	50th	75th	90th	95th	98th
< 1 month	33.60	42.10	51.30	70.60	99.60	138.90	185.70	220.00	265.60
1 to 3 months*	33.40	42.00	51.10	70.40	99.40	138.60	185.30	219.60	265.10
3 to 6 months	33.20	41.70	50.80	70.00	98.80	137.90	184.50	218.70	264.10
6 to 12 months	32.90	41.30	50.30	69.40	98.10	136.90	183.30	217.40	262.70
1 to 3 years	31.80	40.00	48.80	67.40	95.40	133.60	179.30	213.10	258.00
3 to 5 years	30.00	37.80	46.30	64.20	91.30	128.30	173.00	206.10	250.40
5 to 8 years	27.80	35.20	43.20	60.20	86.00	121.50	164.70	197.00	240.50
8 to 12 years	24.70	31.40	38.70	54.40	78.30	111.50	152.40	183.40	225.70
12 to 16 years	21.10	27.10	33.70	47.70	69.30	99.60	137.60	167.10	208.00
16 to 20 years	17.80	23.20	29.00	41.50	60.70	88.10	123.20	151.10	190.90
20 to 30 years	13.90	18.20	22.80	32.80	48.50	71.30	101.30	125.60	161.40
30 to 40 years	11.00	14.10	17.50	25.20	37.50	55.80	80.30	100.20	129.20
40 to 50 years	8.80	11.30	14.00	20.10	30.40	46.20	68.20	86.50	113.70
50 to 60 years	6.90	8.80	11.00	15.90	24.40	38.20	58.40	76.00	103.40
60 to 70 years	5.60	7.10	8.90	13.00	20.40	33.20	53.40	72.50	104.80
70 to 80 years	4.70	6.00	7.50	11.10	17.80	30.40	52.50	76.00	120.90
80 to 90 years	3.90	5.00	6.30	9.40	15.60	28.10	53.40	84.80	158.30

*The term "to" specifies the upper limit in the sense of "less than".

Table S3. Percentiles of heart-rate corrected SDNN (in milliseconds) for women.

Age group	2nd	5th	10th	25th	50th	75th	90th	95th	98th
< 1 month	35.10	44.90	55.40	77.20	109.20	151.40	200.40	235.90	282.20
1 to 3 months*	35.00	44.80	55.20	77.00	108.80	150.90	199.90	235.30	281.60
3 to 6 months	34.70	44.50	54.80	76.40	108.10	150.10	198.80	234.10	280.30
6 to 12 months	34.30	44.00	54.20	75.70	107.10	148.70	197.20	232.30	278.30
1 to 3 years	33.10	42.40	52.40	73.20	103.80	144.40	191.90	226.50	271.90
3 to 5 years	31.20	40.00	49.50	69.30	98.60	137.70	183.70	217.40	261.90
5 to 8 years	28.90	37.10	46.00	64.60	92.30	129.60	173.80	206.40	249.80
8 to 12 years	25.80	33.40	41.50	58.50	84.00	118.70	160.50	191.60	233.50
12 to 16 years	22.70	29.40	36.70	52.00	75.20	107.00	146.00	175.50	215.70
16 to 20 years	20.00	26.10	32.50	46.30	67.30	96.50	132.70	160.60	199.20
20 to 30 years	16.60	21.60	26.90	38.40	56.00	80.90	112.60	137.40	172.70
30 to 40 years	13.30	17.10	21.10	29.90	43.40	62.90	88.30	108.60	137.80
40 to 50 years	10.60	13.40	16.40	23.00	33.30	48.50	68.70	85.20	109.50
50 to 60 years	8.40	10.40	12.70	17.60	25.60	37.80	54.50	68.70	90.20
60 to 70 years	6.90	8.50	10.30	14.20	20.70	31.20	46.50	60.30	82.80
70 to 80 years	5.90	7.30	8.80	12.20	17.90	27.60	43.50	59.50	89.50
80 to 90 years	5.10	6.40	7.80	10.90	16.10	25.80	44.20	67.40	126.10

*The term "to" specifies the upper limit in the sense of "less than".

Table S4. Percentiles of heart-rate corrected RMSSD (in milliseconds) for men.

Age group	2nd	5th	10th	25th	50th	75th	90th	95th	98th
< 1 month	53.00	66.00	79.80	108.90	153.10	214.90	293.00	354.00	440.20
1 to 3 months*	52.70	65.60	79.40	108.40	152.40	214.00	291.80	352.70	438.70
3 to 6 months	52.10	64.90	78.60	107.30	150.90	212.10	289.50	350.00	435.70
6 to 12 months	51.20	63.90	77.40	105.80	148.80	209.40	286.00	346.10	431.10
1 to 3 years	48.40	60.50	73.40	100.60	141.90	200.30	274.50	333.00	416.30
3 to 5 years	44.10	55.40	67.40	92.70	131.40	186.30	256.70	312.70	393.10
5 to 8 years	39.10	49.30	60.30	83.40	118.80	169.50	235.10	287.90	364.60
8 to 12 years	32.80	41.60	51.10	71.20	102.10	146.80	205.70	253.80	324.90
12 to 16 years	26.50	33.90	41.90	58.70	84.80	122.80	173.80	216.30	280.30
16 to 20 years	21.60	27.70	34.30	48.30	70.10	102.10	145.70	182.60	239.30
20 to 30 years	16.00	20.60	25.40	35.80	51.90	75.80	109.10	137.70	182.70
30 to 40 years	12.10	15.40	18.80	26.20	37.70	55.10	79.40	100.70	134.40
40 to 50 years	9.80	12.30	15.00	20.80	29.90	43.90	64.00	82.00	111.50
50 to 60 years	7.70	9.70	11.90	16.60	24.10	36.00	54.20	71.60	102.50
60 to 70 years	6.20	8.00	9.90	14.00	20.70	32.00	51.00	71.60	114.60
70 to 80 years	5.40	7.00	8.80	12.60	19.00	30.60	52.90	81.50	157.10
80 to 90 years	4.90	6.40	8.10	11.70	17.90	29.80	56.20	96.20	230.10

*The term “to” specifies the upper limit in the sense of “less than”.

Table S5. Percentiles of heart-rate corrected RMSSD (in milliseconds) for women.

Age group	2nd	5th	10th	25th	50th	75th	90th	95th	98th
< 1 month	56.9	70.8	85.5	116.1	161.9	225.9	307.2	371.6	463.9
1 to 3 months*	56.6	70.5	85.1	115.5	161.1	224.9	305.9	370.1	462.2
3 to 6 months	56.0	69.7	84.2	114.4	159.6	222.8	303.3	367.0	458.6
6 to 12 months	55.1	68.6	82.9	112.7	157.3	219.8	299.4	362.5	453.2
1 to 3 years	52.1	65.0	78.7	107.2	150.0	210.0	286.7	347.7	435.8
3 to 5 years	47.6	59.7	72.4	98.9	138.9	195.1	267.3	325.2	409.4
5 to 8 years	42.5	53.5	65.1	89.4	126.0	177.8	244.8	298.9	378.3
8 to 12 years	36.1	45.9	56.1	77.5	109.7	155.7	215.8	265.0	338.1
12 to 16 years	30.1	38.5	47.3	65.7	93.6	133.6	186.7	230.7	297.1
16 to 20 years	25.3	32.6	40.3	56.2	80.4	115.3	162.1	201.5	261.8
20 to 30 years	19.8	25.6	31.7	44.5	63.7	91.6	129.5	162.0	212.9
30 to 40 years	15.3	19.7	24.2	33.6	47.7	68.2	96.2	120.3	158.4
40 to 50 years	12.1	15.3	18.6	25.4	35.8	50.8	71.5	89.6	118.5
50 to 60 years	9.5	11.9	14.4	19.5	27.3	38.9	55.5	70.5	95.6
60 to 70 years	8.0	9.9	11.9	16.1	22.6	32.7	48.2	63.6	92.2
70 to 80 years	7.0	8.8	10.6	14.4	20.3	30.2	47.2	66.9	112.1
80 to 90 years	6.3	8.1	9.8	13.5	19.2	29.3	49.7	78.4	166.7

*The term “to” specifies the upper limit in the sense of “less than”.
